# Health Human Resources Guidelines: Minimum Staffing Standards and Role Descriptions for Canadian Cystic Fibrosis Healthcare Teams

**DOI:** 10.1155/2016/6369704

**Published:** 2016-04-17

**Authors:** Ian D. McIntosh

**Affiliations:** Cystic Fibrosis Canada, Toronto, ON, Canada M4C 2P9

## Abstract

In cystic fibrosis clinics across Canada, the most common barrier that healthcare workers face when providing care to their patients is having too little time. The Health Human Resources Guidelines were developed to define specifically what amounts of time should be allocated for each discipline of cystic fibrosis clinical care and to provide a description of all the roles involved, reinforcing how these work together to provide comprehensive multidisciplinary care. With involvement from all cystic fibrosis clinics in Canada, through the use of a tailored survey, the Health Human Resources Guidelines are an exclusively Canadian document that has been developed for implementation across the country. The guidelines have been incorporated into a national Accreditation Site Visit program for use in evaluating and improving care across the country and have been distributed to all Canadian cystic fibrosis clinics. The guidelines provide hospital administrators with clear benchmarks for allocating personnel resources to the cystic fibrosis clinics hosted within their institutions.

## 1. Introduction

The Health Human Resources Guidelines (HHRG) have been developed, in collaboration with medical and clinical advisors, to ensure that all Canadian cystic fibrosis (CF) clinics are able to operate with adequate personnel resources. The goal is to provide the best care possible to Canadians with CF and their families.

The development and adoption of guidelines for use across the country provide clinics with documented requirements with which to approach their hospital administrators, to illustrate and support the need for more resources. It must be emphasized that these are* minimum standards*.

With that being said, the HHRG should be considered only as* guidelines*. If a particular staffing complement is currently higher than the nationally suggested minimum, it should not be reduced or restricted in light of these guidelines. Local circumstances must prevail.

With involvement from leading authorities on CF clinical care in Canada and participation from clinics across the country and in consultation with numerous clinicians who treat those with cystic fibrosis on a daily basis, these guidelines are a fully representative and exclusively Canadian initiative.

*The objective of this document* is to provide a* measurement tool* for use when accrediting and evaluating each clinic's resources and services and to provide* advocacy support* to help obtain these resources.

## 2. Methods: A Canadian Perspective—How the Country Contributed to These Guidelines

A survey was developed to determine from clinics the number of patients served and what current CF clinic staffing allocations are available at their respective centres. By contrast, they were asked what allocations are realistically required. All staff disciplines included in CF care were listed, and respondents were requested to indicate the allocated full-time equivalent (FTE) at their clinic for each discipline (these are displayed as Current and Optimal, in Supplementary Information: Survey results; see Supplementary Material available online at http://dx.doi.org/10.1155/2016/6369704).

The survey explained that 1.0 FTE equals five full-time days per week, protected for inpatient and/or outpatient CF care. Using this measurement, all staff complements across the country could be compared equally.

The survey was posted on SurveyMonkey.com and an e-mail message was sent to clinic directors and coordinators of all CF clinics in Canada, requesting their participation and explaining the purpose and website address of the survey. English and French versions of the survey were available, and a two-week turnaround deadline was given.

The initial response rate to the survey was high; however, it was important to ensure that all clinics had a representative voice in the process. In a second wave, all nonresponders were contacted and reminded to complete the survey. A third and final effort by telephone, fax, and in person was successful in soliciting responses from all remaining nonresponders. Ultimately, every CF clinic in Canada participated in the development of these guidelines.

## 3. Results

Responses were received from all 42 CF clinics in Canada, submitted from 12 nurses; 13 clinic directors; 3 clinic directors along with other team members together; and 10 respondents who did not identify themselves. Four responses were received jointly from separate paediatric and adult clinics that are located at the same site.

Responses for staffing allocations were entered into a spreadsheet. In many cases, FTE allocations had to be interpreted and calculated, in order to enable standardized comparisons; however, two clinics were unable to specify allocated full-time equivalents and therefore were not included.

Using population sizes, the results were sorted according to existing groupings that reflect acknowledged limits at which the population load requires additional human resources: ≤20 patients, 21–75 patients, 76–150 patients, 151–249 patients, and 250+ patients. For each staff discipline within each range, minimum, maximum, and mean FTE complements were determined.

The resulting recommended FTE allocations do not necessarily increase along with the population size groupings, primarily as a result of the small sample size of responding clinics in certain groupings. Regardless, the numbers presented reflect the information provided by the clinics themselves and were not altered; any adjustments would have been arbitrary and, in order not to compromise credibility, none were made.

Descriptions of the multidisciplinary roles were developed based on extensive knowledge of the Canadian CF clinic landscape, obtained during visits to all clinics over a multiyear period, and on advisement of on-site physicians and clinicians.

Initial drafts of this document were circulated amongst the former Clinic Subcommittee of Cystic Fibrosis Canada's Medical/Scientific Advisory Committee; the current Healthcare Advisory Council; the Nursing Advisory Group; the Board of Directors; and other vested opinion leaders for review, evaluation, and comment. Adjustments were made accordingly; however, comments affected only the role descriptions, not the specific FTE allocations.

## 4. Discussion

### 4.1. Principles of CF Care

Cystic fibrosis clinics provide specialized multidisciplinary care. In Canada, there are outpatient CF clinics in hospitals in most major cities, and when hospitalization is required, CF individuals ideally are admitted to inpatient facilities that are part of the same institution.

In cities where there are separate paediatric and adult clinics, there is usually a close relationship between the two services, and interaction amongst the multidisciplinary counterparts is encouraged, to ensure continuity of care and a smoother transition process when the transfer is made from paediatric to adult care.

*Patients see various healthcare professionals at each clinic visit*. This multidisciplinary approach optimizes the care delivered and promotes a long-term association with CF clinical care. As cystic fibrosis is a multisystem disease, each healthcare team member has a* specific area of expertise*. CF must be treated throughout life, and it is important that affected individuals develop a comfortable, trusting relationship with all clinic personnel.

CF clinics take place according to a regular schedule, and those with CF should* attend clinic once every three or four months*, at a minimum. Additional visits should take place as necessary, and individual team members should be available for consultation outside of clinic times.

### 4.2. CF Clinics

#### 4.2.1. Institutional Support

Clinics are supported by their host institutions, and often members of the CF team are loaned to the clinic; for example, the physiotherapy department may designate one physiotherapist to work specifically with the CF population and to participate in all CF-related activities.

The most common consideration in allocating staff resources is how much money is available to support each team member. In other circumstances, actual availability of personnel is a deciding factor; for example, if a psychologist is not available within the institution or community, no amount of money will ensure such an individual's involvement in CF care, although this does not preclude the need for this service.

#### 4.2.2. Accreditation

CF clinics participate in a national* Accreditation Site Visit program*.

By hosting an Accreditation Site Visit, medical and clinical personnel at a clinic are able to discuss their roles, treatment protocols, and limitations, with medical and clinical leaders from CF clinics in other centres. This information exchange strengthens the Canadian CF clinical network and ensures that care is consistent across the country. Following each visit, recommendations are provided to improve and enhance all aspects of every clinic's services.

These Health Human Resources Guidelines have been developed to ensure adequate and consistent standards across Canada and will serve as* benchmarks* by which clinical services are evaluated, during each Accreditation Site Visit. With a recognized set of Canadian standards, clinics—and their administrators—are expected to justify staffing levels, where deficiencies exist.

#### 4.2.3. A CF Clinic by Definition

A clinic is both a physical space and a structured arrangement for the delivery of care.

Along with the* required healthcare workers*, a CF clinic must have* appropriate physical space* in which to function. There must be adequate rooms in which to meet with and examine patients. For infection control purposes, waiting rooms are not recommended—to minimize patient interaction—but the clinic area should include a place where team members may hold pre- and postclinic meetings and conduct administrative tasks. There must be adequate facilities for patient files and charts.

There must be unrestricted* access to hospital beds* for admissions, and facilities must observe infection control protocols. Typically, the CF clinic coordinator, in collaboration with ward nurses, ensures continuity of care.

*Laboratory and testing facilities* must be available, locally or on referral, including pulmonary function testing equipment, such as plethysmographs and portable spirometers; microbiological testing facilities, to identify important pathogens; CF diagnostic sweat chloride testing; and genetic testing and counselling. And every clinic should establish lines of communication with a lung transplant program for referrals.

In addition to* infection control policies* in place at every institution, CF clinics must have specific policies to reduce cross-infection of pathogens that are of concern to the CF population. Specifically, procedures must be in place to ensure limited interaction amongst patients—for example, through staggered clinic scheduling; isolated clinic examination rooms; and visits outside of clinic times—along with procedures for clinic personnel, inpatient nursing staff, and family members. Infection control policies must be developed and enforced, and all those associated with the clinic should have a full understanding of the implications of good infection control.

As an extension of its services, each clinic is encouraged to participate in* research initiatives and clinical trials* and is expected to participate in the development, testing, and/or discussion of new medications and treatments.

### 4.3. Multidisciplinary Care: A Team Approach

As a multisystem disease with a* proven model of multidisciplinary care*, all components of the CF clinic must be available, to ensure that comprehensive care is delivered. Patient care becomes compromised if sufficient access to services is not available.

A* CF healthcare team is comprised of several professionals*, each with specific expertise. A combined paediatric/adult clinic may share healthcare professionals, but not always; certain health issues, though common in CF, differ with age: learning to swallow pills is different from addressing osteoporosis.

Likewise, inpatient and outpatient care are best delivered by the same individuals, to ensure consistency of care and to reinforce principles of care. When team members are not able to see both outpatients and inpatients, collaborative communication amongst their counterparts is encouraged to ensure consistency.

Availability of personnel and support within each institution varies across the country, but there are* minimum staffing requirements* for CF care to ensure that it is delivered adequately. These Health Human Resources Guidelines were developed so that clinics and institutions are aware of the* necessary components of CF clinical care*.

Where possible, a recommended guideline is indicated in Tables 1 [Table tab2] [Table tab3] [Table tab4] [Table tab5] [Table tab6] [Table tab7] [Table tab8]–9 for the minimum staffing standard for each individual role; this includes all inpatient and outpatient involvement, with no distinction between paediatric and adult care. These guidelines have been developed considering current availability—and current need—from clinics across the country and comprise* a standard that is acknowledged across the country*.

### 4.4. Role Descriptions for CF Healthcare Teams

#### 4.4.1. Primary Personnel

The following multidisciplinary team members make up the core of a CF clinic.

*Clinic Director/CF Physician*. The clinic director, or medical director, is the* physician responsible for the medical aspects of care*, making all medical decisions and referrals. At paediatric clinics, this physician often initiates and/or confirms the diagnosis of CF, typically in collaboration with a provincial newborn screening program. The clinic director outlines patients' medical profiles, prescribes medications, oversees treatments administered by the healthcare team, and can admit patients when necessary.

The clinic director, or designated physician, sees CF individuals at every clinic visit and is available for consultation outside of clinic times. Few physicians are involved with CF exclusively, but many devote a significant portion of time to CF, often including involvement with CF research initiatives.

As a leadership role, the clinic director selects clinic personnel and develops clinic policies. Ultimately, a minimum physician commitment is required in order for a CF clinic to function adequately.

Oftentimes a clinic director is a respirologist; however, other subspecialist physicians may also assume this role, including infectious disease physicians, gastroenterologists, and paediatricians.

In larger CF clinics, the clinic director works with one or more associated CF physicians, who share the medical load and provide backup medical support (see Table 1 for recommended minimum staffing standards).

*Nurse Coordinator/Clinic Coordinator*. The clinic coordinator, often a registered nurse or nurse practitioner, plays a pivotal role* coordinating the healthcare team* and providing continuity between outpatient and inpatient services. The coordinator often recognizes and alerts other team members to changes in patients' psychological and medical conditions and is a support for patients and family members, explaining diagnosis, hospitalization, or changes in therapy.

The coordinator* organizes and formulates treatment regimens* developed by the physician and other team members, ensures that procedures and therapies are understood and promoted, and is commonly the first point of contact for patients and families with questions.

Encouraging communication amongst the clinic, hospital care teams, patients, and families, the coordinator provides* education* to all parties and to other medical and healthcare professionals and lay groups (see Table 2 for recommended minimum staffing standards).

*Dietitian/Nutritionist*. The dietitian monitors patients'* nutritional status* and encourages appropriate dietary habits, constructing specialized diets and working with patients and families to optimize nutritional health. The dietitian recommends nutritional supplements and offers advice on preparation of meals, high fat recipes, and additional dietary resources.

The dietitian instructs on the use of pancreatic enzymes: what dosage to take with meals and snacks to derive the most benefit.

The dietitian works with a gastroenterologist if there are complications such as malnutrition. In extreme cases of nutritional deprivation where oral intake is inadequate, tube feeding may be prescribed and is monitored by the dietitian and gastroenterologist.

Increasingly, the dietitian is involved in the management of CF-related diabetes, serving as a diabetes educator, often in collaboration with nursing personnel (see Table 3 for recommended minimum staffing standards).

*Physiotherapist*. The physiotherapist educates individuals and families about* physical airway clearance techniques*. The physiotherapist sees CF individuals during clinic, administers physiotherapy sessions for both hospitalized and ambulatory patients, and should be available outside of clinic times for assistance.

There are several methods of physiotherapy available—manual and mechanical—and the physiotherapist will tailor regimens to individuals' needs. The physiotherapist will vary or rearrange procedures or introduce new methods, to add variety and help encourage adherence.

Once appropriate techniques have been taught, the physiotherapist monitors manoeuvres and makes adjustments to maintain and improve effectiveness.

The physiotherapist stays abreast of new techniques and devices, as well as individuals' responses to such new approaches. The physiotherapist is knowledgeable about provincial coverage for equipment and can provide direction for those seeking financial assistance (see Table 4 for recommended minimum staffing standards).

*Social Worker*. The social worker plays an important role addressing* psychosocial issues* and* financial issues*.

As the main resource person available to those with psychosocial issues, the social worker provides counselling most often regarding diagnosis of CF and subsequent lifestyle changes required for optimum health, adherence to prescribed drug and physical therapies, and living with a chronic disease. Social workers can also be involved with counselling and referral on a variety of topics from career decisions to sexuality.

The social worker may refer to and liaise with a psychologist or psychiatrist for additional counselling support.

The social worker also helps address financial concerns. Being acquainted with programs for coverage of drugs, equipment, and oxygen and with private insurance plans, the social worker offers assistance for accessing these resources. Also, the social worker is instrumental in arranging social assistance and may counsel on financial matters (see Table 5 for recommended minimum staffing standards).

*Pharmacist*. The pharmacy is responsible for dispensing* drugs, vitamins, enzymes*, and, in many cases,* nutritional supplements*. Drugs should be available for CF individuals to pick up at clinic, and many pharmacies send medications to distant individuals or families.

A pharmacist who works closely with the CF clinic is familiar with the disease and can provide advice on drug therapy and other types of education, to patients, families, and healthcare workers. Similarly, a designated CF pharmacist can educate on the purpose of each drug, the dosage, and routine for administration. A pharmacist can caution about interactions and adverse effects and can teach about new medications.

When drug use is tracked, the pharmacist can monitor adherence to drug therapy and can ensure that costs are kept to a minimum—for patients, the institution, and the provincial government.

The pharmacist can also provide information about provincial drug coverage programs and how financial coverage can be obtained (see Table 6 for recommended minimum staffing standards).

*CF Clinic Secretary/Administrator*. A designated clinic secretary conducts the administrative tasks for the clinic, such as correspondence, filing, dictation, booking appointments, and organizing charts. In some cases, the CF clinic secretary also arranges referrals to other healthcare team members and to services in the hospital, for example, the diabetes clinic or allergy clinic, or specific laboratories.

The CF clinic secretary/administrator may also be responsible for the preparation and submission of data to the Canadian Cystic Fibrosis Registry, often in tandem with the clinic coordinator (see Table 7 for recommended minimum staffing standards).

*Respiratory Technician*. A respiratory technician conducts* lung function measurement testing*, tracks test results, and recognizes warning signs when lung functions decline. Where respiratory laboratories produce graphic representation of patients' longitudinal lung function status, the clinic director and others may use this to evaluate patient history and progress.

It is advantageous to have the same individual performing all procedures and tests—these procedures are often done at every clinic visit, and consistency of results is crucial. For the paediatric CF population, establishing a relationship with the respiratory technician can help to alleviate the fear of performing the tests and can help ensure usable responses (see Table 8 for recommended minimum staffing standards).

*Psychiatrist or Psychologist*. When special psychosocial concerns arise, a medical professional specializing in* mental health* should be available, and referral to a psychiatrist or psychologist will often be made.

A psychiatrist or psychologist is available for consultation any time, often outside of clinic times, but generally works in conjunction with the CF healthcare team members (see Table 9 for recommended minimum staffing standards).

#### 4.4.2. Additional Personnel

Many other key roles comprise a complete CF healthcare team, though often on a less frequent basis—involvement can depend on the size of the clinic and the local resources that are available. These are important roles for inclusion in a discussion of Canadian CF multidisciplinary clinical care, but not every clinic has these roles, or with consistency. As such, specific FTE standards are not provided below, but rather only explanations of the roles, to illustrate their breadth and importance. These include the following.

*CF Clinic Nurse*. In addition to the clinic coordinator, some CF clinics have a* designated CF clinic nurse*, who provides additional nursing support. A clinic nurse provides continuity of care when the coordinator is unavailable and additional support during busy clinics and often concentrates on one component of the clinic's operations.

*Other Medical Subspecialists*. Subspecialists—or physicians with a specialized focus of medical care—are commonly associated with CF clinical care. Access can be either on referral or more formally if the physician attends outpatient CF clinics periodically.

Subspecialists most typically involved in CF care may include gastroenterologists, endocrinologists, otolaryngologists, and infectious disease specialists.

When a formal attachment is developed with a CF clinic, a subspecialist can become familiar with the patients and with the complications of the disease itself.

Availability of certain subspecialists may be limited—for example, not every clinic may have access to a paediatric gastroenterologist—and it may be necessary to make referrals outside of the institution.

*Child Life Worker*. The child life worker primarily* counsels young children*. Working in ways best understood by a child, issues of pain, daily therapy routines, and death are addressed. The format of a child life worker's therapy is often play, using games and crafts that are more relatable to a child, and that can help to normalize a hospital experience.

*Chaplain*. A chaplain or other* spiritual counsellor* may provide comfort during times of death and dying. Family, friends, and other CF team members may need* emotional support* during times of grief, and a designated chaplain who is familiar with the disease is better able to provide personalized support.

A chaplain may offer counselling of a more religious direction, when appropriate circumstances arise.

## Supplementary Material

Survey results: Survey results include information reported from CF clinics in Canada, and provide details – by discipline and acknowledged population size ranges – of what full-time equivalent complements of time are currently in place (Current) at each clinic, and what FTEs are realistically required (Optimal). For each discipline within each population size range, minimum, maximum, and mean FTE complements are displayed.
Guidelines from other jurisdictions: To offer international comparisons, links to guidelines from other countries are provided; however, with differences in national healthcare structures, medical facilities, availability of resources, etc., direct comparisons may not reflect the Canadian perspective, and in fact, certain staffing allocations appear extreme compared with Canadian counterparts.

## Figures and Tables

**Table 1:** Clinic director/CF physician.

Number of patients	Full-time equivalent		Total
	Clinic director	CF physician	
≤20	0.18	0.02	0.2
21–75	0.29	0.25	0.54
76–150	0.63	0.61	1.24
151–249	0.5	1.67	2.17
250+	1.0	2.83	3.83

**Table 2 tab2:** Nurse coordinator/clinic coordinator.

Number of patients	Full-time equivalent
≤20	0.4
21–75	0.78
76–150	1.53
151–249	2.5
250+	3.17

**Table 3 tab3:** Dietitian/nutritionist.

Number of patients	Full-time equivalent
≤20	0.15
21–75	0.28
76–150	0.59
151–249	1.0
250+	1.67

**Table 4 tab4:** Physiotherapist.

Number of patients	Full-time equivalent
≤20	0.15
21–75	0.3
76–150	0.59
151–249	1.15
250+	1.83

**Table 5 tab5:** Social worker.

Number of patients	Full-time equivalent
≤20	0.15
21–75	0.3
76–150	0.53
151–249	1.5
250+	1.33

**Table 6 tab6:** Pharmacist.

Number of patients	Full-time equivalent
≤20	0.12
21–75	0.17
76–150	0.31
151–249	0.88
250+	0.73

**Table 7 tab7:** CF clinic secretary/administrator.

Number of patients	Full-time equivalent
≤20	0.13
21–75	0.22
76–150	0.61
151–249	1.00
250+	2.00

**Table 8 tab8:** Respiratory technician.

Number of patients	Full-time equivalent
≤20	0.08
21–75	0.12
76–150	0.29
151–249	0.40
250+	1.00

**Table 9:** Psychiatrist or psychologist.

Number of patients	Full-time equivalent
≤20	0.12
21–75	0.14
76–150	0.21
151–249	0.10
250+	0.33

