# Supplementary material for: Health Human Resources Guidelines: Minimum Staffing Standards and Role Descriptions for Canadian Cystic Fibrosis Healthcare Teams
Source: Can Respir J. 2016 Apr 17;2016:6369704. doi: 10.1155/2016/6369704 (PMC4904508; doi:10.1155/2016/6369704)
Supplement: Supplementary file 1 — Survey results: Survey results include information reported from CF clinics in Canada, and provide details – by discipline and acknowledged population size ranges – of what full-time equivalent complements of time are currently in place (Current) at each clinic, and what FTEs are realistically required (Optimal). For each discipline within each population size range, minimum, maximum, and mean FTE complements are displayed. Guidelines from other jurisdictions: To offer international comparisons, links to guidelines from other countries are provided; however, with differences in national healthcare structures, medical facilities, availability of resources, etc., direct comparisons may not reflect the Canadian perspective, and in fact, certain staffing allocations appear extreme compared with Canadian counterparts. [file 6369704.f1.pdf]

1. Supplementary Information: Survey results*

CURRENT STAFFING LEVELS

Patient Population	Number of Clinics responding**	Clinic Director		Clinic Physician		Physician Total		Nurse Total		Dietitian		Physiotherapist		Social Worker		Pharmacist		Respiratory Therapist		Psychologist		Secretary	
		MIN/MAX	AVG	MIN/MAX	AVG	MIN/MAX	AVG	MIN/MAX	AVG	MIN/MAX	AVG	MIN/MAX	AVG	MIN/MAX	AVG	MIN/MAX	AVG	MIN/MAX	AVG	MIN/MAX	AVG	MIN/MAX	AVG
<20	3	0.05/0.3	0.15	0/0.05	0.02	0.1/0.3	0.17	0.05/0.5	0.25	0.05/0.2	0.12	0.05/0.2	0.12	0.05/0.15	0.10	0.05/0.2	0.10	0/0.1	0.05	0/0.1	0.03	0.05/0.1	0.07
21-75	12	0/1	0.20	0/1	0.16	0/2	0.36	0/1.1	0.52	0.05/0.4	0.14	0/1.1	0.19	0/0.5	0.15	0/0.15	0.04	0/0.25	0.08	0/0	0.00	0/0.2	0.04
76-150	15	0.1/1	0.53	0/2	0.41	0.15/3	0.94	0.4/2.1	1.01	0/0.8	0.37	0/0.5	0.28	0/0.7	0.30	0/0.6	0.13	0/0.4	0.11	0/0.4	0.06	0/1	0.34
151-249	2	0.2/0.5	0.35	0/2.33	1.17	0.2/2.83	1.52	1/1	1.00	0.6/0.8	0.70	0.8/1	0.90	1/1	1.00	0/1	0.50	0/0.3	0.15	0/0	0.00	0.9/1	0.95
>250	3	0.5/1	0.67	0.75/3.5	1.92	1.25/4.5	2.58	2/3	2.33	0.8/1.8	1.37	0.4/2	1.30	0.8/1	0.93	0/1	0.40	0/1.8	0.60	0/0.3	0.10	1/2	1.33

OPTIMAL STAFFING LEVELS

Patient Population	Number of Clinics responding**	Clinic Director		Clinic Physician		Total Physician		Nurse Total		Dietitian		Physiotherapist		Social Worker		Pharmacist		Respiratory Therapist		Psychologist		Secretary	
		MIN/MAX	AVG	MIN/MAX	AVG	MIN/MAX	AVG	MIN/MAX	AVG	MIN/MAX	AVG	MIN/MAX	AVG	MIN/MAX	AVG	MIN/MAX	AVG	MIN/MAX	AVG	MIN/MAX	AVG	MIN/MAX	AVG
<20	3	0.05/0.3	0.18	0/0.05	0.02	0.1/0.3	0.20	0.1/0.6	0.40	0.05/0.3	0.15	0.05/0.3	0.15	0.05/0.2	0.15	0.05/0.2	0.12	0/0.2	0.08	0.05/0.2	0.12	0.1/0.2	0.13
21-75	12	0/1	0.29	0/1	0.25	0/2	0.54	0/2	0.78	0.05/0.8	0.28	0/1.25	0.30	0/1	0.30	0.05/0.5	0.17	0/0.5	0.12	0/0.5	0.14	0/0.75	0.22
76-150	15	0.1/1	0.63	0/2	0.61	0.3/3	1.24	1/4	1.53	0/1.5	0.59	0/2	0.59	0/1	0.53	0/1	0.31	0/1	0.29	0/0.5	0.21	0/1.2	0.61
151-249	2	0.5/0.5	0.50	1/2.33	1.67	1.5/2.83	2.17	2/3	2.50	1/1	1.00	0.8/1.5	1.15	1.5/1.5	1.50	0.75/1	0.88	0.3/0.5	0.40	0/0.2	0.10	1/1	1.00
>250	3	1/1	1.00	2/3.5	2.83	3/4.5	3.83	2/4.5	3.17	1/2.5	1.67	1/3	1.83	1/1.5	1.33	0.5/1	0.73	0/2	1.00	0/0.5	0.33	1.5/2.5	2.00

* Based on survey results provided from Canadian CF clinics; information current as of 2010.

** Does not include incomplete or unusable information.

2. Supplementary Information: Guidelines from other jurisdictions

To offer international comparisons, links to guidelines from other countries are listed below; however, with differences in national healthcare structures, medical facilities, availability of resources and so on, direct comparisons may not reflect the Canadian perspective, and in fact, certain staffing allocations seem extreme compared to Canadian counterparts. For example, for the role of CF physiotherapist, the guidelines from both Australia and the UK suggest full-time equivalents more than double those of the Canadian guidelines; and those from Europe suggest that nursing roles should be double those recommended in Canada.

- Australia

http://www.thoracic.org.au/imagesDB/wysiwyg/CF_standardsofcare_Australia_2008.pdf

- Europe

http://www.elsevier.com/framework_products/promis_misc/2005.pdf

- New Zealand

http://www.thoracic.org.au/imagesDB/wysiwyg/CF_StandardsofCare_NZ2010.pdf

- United Kingdom

http://www.cysticfibrosis.org.uk/media/448939/cd-standards-of-care-dec-2011.pdf

- USA

Infant Care (Abstract only)
http://www.jpeds.com/article/S0022-3476(09)00881-6/abstract

Adult care
http://www.cff.org/UploadedFiles/treatments/CFCareGuidelines/AgeSpecificCare/CF-Adult-Care-Chest-2004.pdf
